# Evaluation of Cancer Metabolomics Using *ex vivo* High Resolution Magic Angle Spinning (HRMAS) Magnetic Resonance Spectroscopy (MRS)

**DOI:** 10.3390/metabo6010011

**Published:** 2016-03-22

**Authors:** Taylor L. Fuss, Leo L. Cheng

**Affiliations:** Departments of Radiology and Pathology, Massachusetts General Hospital, Harvard Medical School, 149 13th Street, CNY-6, Charlestown, MA 02129, USA; tfuss@mgh.harvard.edu

**Keywords:** metabolomics, high-resolution magic angle spinning (HRMAS), magnetic resonance spectroscopy (MRS), cancer

## Abstract

According to World Health Organization (WHO) estimates, cancer is responsible for more deaths than all coronary heart disease or stroke worldwide, serving as a major public health threat around the world. High resolution magic angle spinning (HRMAS) magnetic resonance spectroscopy (MRS) has demonstrated its usefulness in the identification of cancer metabolic markers with the potential to improve diagnosis and prognosis for the oncology clinic, due partially to its ability to preserve tissue architecture for subsequent histological and molecular pathology analysis. Capable of the quantification of individual metabolites, ratios of metabolites, and entire metabolomic profiles, HRMAS MRS is one of the major techniques now used in cancer metabolomic research. This article reviews and discusses literature reports of HRMAS MRS studies of cancer metabolomics published between 2010 and 2015 according to anatomical origins, including brain, breast, prostate, lung, gastrointestinal, and neuroendocrine cancers. These studies focused on improving diagnosis and understanding patient prognostication, monitoring treatment effects, as well as correlating with the use of *in vivo* MRS in cancer clinics.

## 1. Introduction

Cancer is currently responsible for more deaths than all coronary heart disease or stroke worldwide, serving as a major public health threat worldwide with 14.1 million new cancer cases and 8.2 million cancer deaths in 2012 [1]. With the increased emphasis on the clinical value of early cancer detection and treatment through better understanding of cancer biology, research directed toward the discovery of cancer molecular characteristics has extended into every biological aspect, from earlier investigations of cancer genomics and proteomics to recent efforts in cancer metabolomics. Metabolites are the small molecule intermediates and products of metabolism that together compose the complete metabolome of a biological sample, providing insight into the metabolic processes within. Metabolomics evaluates all of the measureable metabolites and their changes within a metabolome associated with all of the involved physiological and pathological processes, offering a more comprehensive understanding of cancer biology and disease progression that cannot be achieved by the interrogations of any single metabolite or isolated metabolic pathway, and presents improved potential to inform diagnostics and treatment management in the clinic. Metabolomics established itself from the bases of early studies on the quantification of individual metabolites and their role in cancer progression, but expanded its focus to the horizon of entire metabolomic profiles and their relationships with cancer diagnosis and patient prognostication.

Magnetic resonance spectroscopy (MRS), together with mass spectrometry, has been the main driving force behind the development of cancer metabolomics. High resolution magic angle spinning (HRMAS) MRS, with its demonstrated ability to investigate intact tissue metabolites and histology from the same specimens, has contributed immensely towards the formation and development of MRS-based cancer metabolomics. HRMAS methodology was introduced as a powerful tool for the metabolic analysis of intact tissue samples in 1996 [2,3]. Borrowing the solid state MRS line-narrowing concept of magic angle spinning, HRMAS applies a mechanical rotation of the sample at a 54.7° angle from the direction of the magnetic field, and allows for the observation of individual metabolites in the intact tissues from their solution-like high-resolution spectra as demonstrated by many early works reported in the late 1990s [4,5,6,7,8]. The method preserves tissue architectures and allows for further subsequent examinations of histopathology, the current gold standard for cancer diagnosis, after tissue MRS measurements [9]. Independent from morphology-based histology, metabolomic knowledge thus acquired forms another independent informational dimension that is expected to compensate for the evaluations of traditional histopathology with the improved ability to accurately determine biological characteristics of disease and lead to the prediction of disease progression and patient prognostication.

In this review article, we will discuss studies utilizing HRMAS MRS in the evaluation of cancer metabolomics published after 2010, and we organize the review mainly according to cancer types. Readers interested in the early history of HRMAS MRS applications in cancer metabolomics and medicine are encouraged to consult other systematic review articles published previously [10,11,12].

## 2. Brain Cancer

Parallel to the early development of HRMAS methodology for disease evaluations seen with human brain tumors, the majority of HRMAS MRS metabolomics cancer research has also been reported in reference to human brain cancer. Brain cancer is not the most prevalent cancer in adults, with 256,000 estimated new cases and 189,000 estimated deaths in 2012 and the highest incidence and mortality rates in more developed regions such as Australia/New Zealand, Europe, and North America, reflective of expanded access to diagnostic imaging [1]. Nevertheless, since the brain is the most suitable organ for MR evaluations, including MRS, due to its combined status of relative homogeneity in magnetic susceptibility and of its minimal respiratory and cardiac motions compared with other organs, brain tumors are typically detected using MR imaging (MRI), and diagnosed with histopathological examination of surgically removed tissue to determine cancer type and grade. Realizing the potential for the characterization of brain tumors, as well as monitoring their progression and responses to therapy, with *in vivo* ^1^H MRS, research efforts have been focused on *ex vivo* HRMAS MRS for its potential correlation with, and interpretation of, *in vivo* MRS. These research efforts led to logical developments of metabolic profile evaluations through intact tissue analyses, and the formation of the concept of brain cancer metabolomics. In this section, due to the extensive coverage of HRMAS MRS utilizations in brain cancer studies, we will organize our discussions of brain cancer *ex vivo* HRMAS MRS applications in terms of: correlations with *in vivo* MRS; propositions of diagnostic profiles for both adult and pediatric diseases; relationship with detailed histopathology; evaluation of treatment responses; developments of basic research applications for the better understanding of biochemical pathways of brain cancer and its progression; and, finally, development and improvement of HRMAS methods.

**Figure 1:**
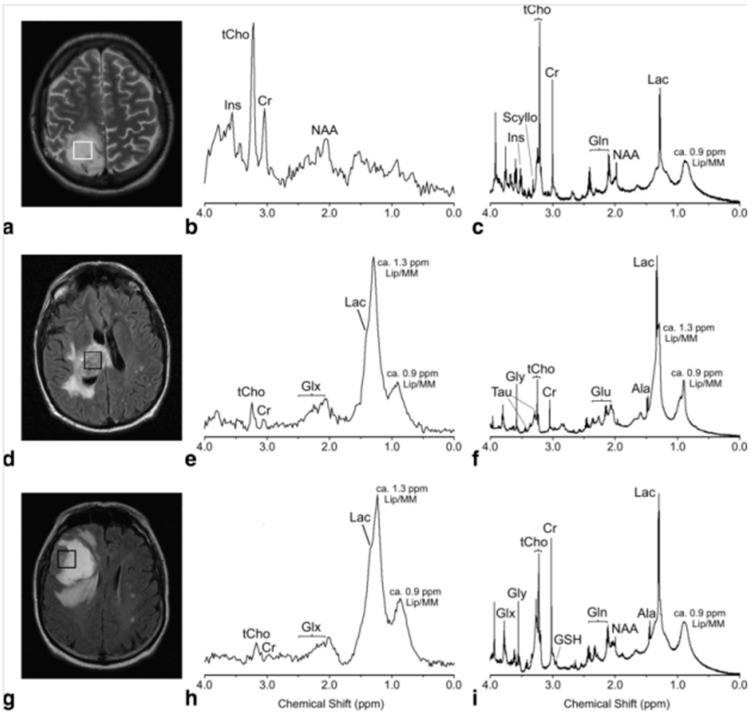
*In vivo* PRESS TE 30 msec spectra (**b**,**e**,**h**) and their equivalent *ex vivo* HRMAS presaturation spectra (**c**,**f**,**i**) from a histopathologically verified astrocytoma grade II (**a**–**c**) and glioblastoma (**d**–**f**) showing unimodal variation of the grayscale pixel values in the voxel placement areas (**a**,**d**), and a histologically verified glioblastoma with multimodal variation of the grayscale pixel values (**g**–**i**). Major metabolite peaks have been labeled, but for clarity not all the metabolites have been labeled in each spectrum (Ala, alanine; Cr, creatine; Gln, glutamine; Glu, glutamate; Glx, (Gln + Glu); Gly, glycine; Ins, *myo*-Inositol; Lac, lactate; Lip/MM, lipids/macromolecules; Scyllo, *scyllo*-Inositol; Tau, taurine; tCho, total cholines) [13].

To characterize human gliomas, the most prominent type of brain tumor in adults, *ex vivo* intact tissue HRMAS MRS has been studied for its relationship with *in vivo* MRS. A 2010 study attempted to correlate *in vivo* Point Resolved Spectroscopy (PRESS) ^1^H MRS data with metabolomic profiles measured from *ex vivo* HRMAS ^1^H MRS for 17 astrocytoma patients [13]. In this study, prior to surgical biopsy, *in vivo* ^1^H MRS was measured on a 1.5T MR scanner, and after *in vivo* evaluations, surgical biopsy was conducted with biopsy tissues measured using HRMAS MRS at 14.1T. The *in vivo* low spectral resolution seen at 1.5T resulted in heavily overlapping signals of glutamate (Glu) and glutamine (Gln), as well as glycerophosphocholine (GPC) and phosphocholine (PChol) (Figure 1). These peaks thus were summed as Glx and tCho in the *in vivo* report. Linear regression analysis revealed significant correlations between *in vivo* and *ex vivo* ^1^H MRS spectra for many major metabolites, such as creatine (Cr), Glx, *myo*-inositol (*m*-Ino), n-acetylaspartate (NAA), *scyllo*-inositol (*s*-Ino), tCho and the lipid/macromolecule (Lip/MM) peaks. Additionally, Cr, *m-*Ino, tCho, and Lip/MM correlated between *in vivo* and *ex vivo* concentrations and presented as important factors in grading astrocytomas. Another study reported using HRMAS MRS to differentiate between World Health Organization grade II and IV (glioblastoma multiforme; GBM) astrocytomas. Analyses of metabolomic profiles with principal component analysis (PCA) were able to clearly distinguish between the two tumor types, with grade II tumors showing increased levels of GPC and *m*-Ino, and GBM showing increased levels of PChol, glycine (Gly), and lipids, as reflected in their clear separation in the principal component (PC)1 *vs.* PC3 score plot [14]. These results suggest the importance of separating GPC and PChol for tumor characterization purposes, and not combining them into tCho, which was, however, impossible to achieve with low-resolution *in vivo* MRS spectra. This observation and caution was further confirmed by comparative studies of metabolomic profiles acquired from transgenic mice with medullablastoma *in vivo* at 7T and *ex vivo* with HRMAS at 11.7T. The results confirm the importance of measuring GPC and PChol individually. The *in vivo* increase in tCho was found to be clearly the result of an increase in PChol only, as GPC remained stable while an observed reduction in choline (Cho) was seen [15].

An HRMAS MRS study evaluated samples from image-guided biopsy acquired from 126 patients of either new or recurrent gliomas of grades II–IV. Using a proportional odds logistic regression model, the study assessed the associations of metabolite parameters with various pathological grades. The results showed high percentages of accuracy in categorizing data from metabolomic profiles according to tumor grades. Classification of primary grade II *vs.* grade IV (GBM) gliomas demonstrated an accuracy of 84%, emphasizing the importance of the *m*-Ino to tCho index (MCI) and 2-hydroxyglutarate (2HG). Classification of primary grade II *vs.* grade III gliomas presented an accuracy of 73%, with significant differences in MCI, phosphoethanolamine (PE), and Gly. Grade III gliomas were distinguished from GBM with 92% accuracy from increased levels of 2HG, tCho, NAA, and Cr/phosphocreatine (PCr) [16]. The progressive reduction in MCI from grade II, to grade III, and to GBM could potentially serve as a marker that can be monitored with *in vivo* MRS for predicting tumor transformation from a lower to a higher grade. These results highlighted the important role of metabolomic profiles in providing more comprehensive diagnostic information, as well as the importance of acquiring profiles with *ex vivo* HRMAS MRS at higher field strength to identify potential biomarkers for clinical use.

*Ex vivo* metabolomic profiles have shown utility in assisting interpretation of *in vivo* MRS spectra for a more accurate diagnosis. A 2012 study acquired metabolomic profiles of intracranial hemangiopericytomas (HPCs), meningothelial meningioma, and peritumoral edematous tissues to improve diagnostic capability of HPCs, a rare tumor whose radiological appearance resembles meningiomas. A direct comparison of HPC *in vivo* MR spectra acquired at 3T and *ex vivo* spectra acquired with HRMAS MRS at 9.4T showed a good agreement**,** while *ex vivo* spectra presented clearer and more distinguishable resonance peaks. It was especially true for the resonance signal of mannitol (Man), an exogenous metabolite produced by the tumoral mass itself to reduce intracranial pressure caused by the presence of the tumor, which appeared as a broad 3T *in vivo* signal at 3.8 ppm; however, at *ex vivo* 9.4T, it presented as multiple signals between 3.6 and 4.0 ppm. As clinical scanner field strength, and thus the spectral resolution, increases, the potential utility of MRS *in vivo* to diagnose and differentiate tumor grades and types increases. This was especially apparent when comparing HPC to meningioma. At 3T, the two *in vivo* spectral profiles were nearly identical. However, in *ex vivo* spectra acquired with HRMAS MRS at 9.4T, there were multiple differences in the metabolomic profiles, including slightly higher lipid levels in HPC. Ratios of *m*-Ino, glucose, and gluthatione with respect to Glu were higher in HPC when compared with meningioma, but ratios of Cr, Gln, alanine (Ala), Gly, and Cho-containing compounds with respect to Glu were lower. Notably, Ala could be detected in metabolomic profiles acquired at 9.4T, but not in those at 3T, and was found in higher levels in meningioma than in HPC [17]. Another study investigating the potential use of ^1^H HRMAS MRS to distinguish between tumor grades from tissue samples of 68 meningiomas found Ala to be a crucial metabolite for differentiation. Analyses using analysis of variance (ANOVA) found absolute concentrations of total Ala and Cr decreased from grade I to grades II and III (*p* < 0.05). The ratio of Gly to Ala correlated significantly to tumor grades (*p* = 0.002), with the mean Gly/Ala value for grade I equal to 0.96 ± 0.31, compared to a mean of 1.8 ± 0.37 for grades II and III, indicating that while Ala and Cr concentrations were lower, the Gly/Ala ratio was higher in histologically aggressive meningiomas. Ala was reported to be the only metabolite with significant indication of primary *vs.* recurring tumor (*p* < 0.05); however, lower mean Cr and Ala concentrations were observed in rapidly recurring tumors when compared with those that did not recur (*p* < 0.001). The Gly/Ala ratio was significantly higher in tumors with invasion than in those without (*p* = 0.02) [18].

In addition to working collaboratively with *in vivo* MRS as a clinical diagnostic tool, HRMAS MRS produced the metabolomic profiles for various tumor types and grades that provided more complete diagnostic profiles for both adult and pediatric brain cancers. Applying one-dimensional (1D) and two-dimensional (2D) HRMAS ^1^H MRS followed by subsequent histopathology for 65 adult brain tumor surgical samples showed that 29 metabolites account for the majority of metabolites shown in the spectra of the main types of brain tumors (astrocytoma grade II, grade III gliomas, GBM, metastases, meningiomas, and lymphomas), and 20 metabolites showed statistically significant differences between tumor types according to PCA that could potentially serve as biomarkers of tumor type. Of particular interest is the significant metabolomic difference between metastasis and GBM while both present a similar imaging appearance on MRI. Positive biomarkers for GBM are seen with Cr, Gly, Gln, and hypo-taurine (h-Tau), while none of them were present as significant factors in the metabolomic profiles of metastasis [19]. Metabolomic profiles of 30 neuroepithelial tumor biopsies, including two grade I astrocytomas, 12 grade II astrocytomas, eight anaplastic grade III astrocytomas, three GBM, and five grade IV medulloblastomas, were also obtained through PCA evaluations of HRMAS ^1^H MRS data. Strong separation was shown between grade I–II and grade III astrocytomas, with regression coefficients indicating levels of NAA, Cr, GPC, and *m*-Ino being higher, and Lac and PChol being lower in grade I–II tumor tissues when compared with those of grade III tumors. Clear separation was also observed between grade I–II and grade IV tumors, with regression coefficients indicating higher levels of Lac, Cr, Cho, and GPC and lower levels of Gly and PChol in grade I–II tumors when compared with grade IV. Grade III and IV tumors also showed separation, with higher levels of Lac, Cr, Cho, GPC and lower levels of *m*-Ino and PChol in grade III compared with grade IV tumors. PCA also showed good separation between GBM and medulloblastomas and between grade I and grade II astrocytomas. Further analysis with ANOVA showed that certain metabolites, including *m*-Ino, Cr, PChol, GPC, Gly, Tau, and Asp, increased or decreased with grade across tumor types [20].

For pediatric brain tumors, PCA was also used on HRMAS MRS data to produce metabolomic profiles from 20 intact tissue samples, including ependymoma, medulloblastoma, and pilocytic astrocytoma. Ependymoma profiles were mostly characterized by intense signals of *m*-Ino, involved in the activation of protein kinase C, which could indicate the grade of tumor. Medulloblastoma spectra were characterized by higher levels of Tau, GPC, PChol, and Cho, all associated with tumor growth and aggressiveness. NAA levels were similar between ependymoma and medulloblastoma. Pilocytic astrocytomas showed a higher concentration of fatty acids [21]. These results confirmed previous suspicions that ependymoma was likely a tumor with biochemical aggressiveness between medulloblastoma and pilocytic astrocytoma, and provided valuable insight into the metabolomic changes of these tumors. *In vivo* MRS profiles acquired at 1.5T were also compared with *ex vivo* profiles acquired using HRMAS MRS at either 11.7T or 14.1T for 15 pediatric biopsy samples. Analysis of matched *in vivo* and *ex vivo* samples found a correlation for the Glu/tCho ratio (*p* = 0.001), and a separate survival analysis of the HRMAS data showed a similar trend to the *in vivo* data, with a higher Glu/tCho ratio inferring worse survival outcomes [22].

HRMAS MRS results showed correlations with histopathology and demonstrated the presence of typical metabolomic patterns emerging from specific histopathological tissue features. HRMAS MRS investigations of biochemical differences between necrosis, high cellularity, and cancer infiltration were carried out with tissue samples from 52 patients of different types of glial tumors, including GBM, and gliomas grade III and grade II. Cluster analysis was used to determine the potential of metabolomic data sets for predicting histopathological tissue features. Three major clusters were identified as tumor margin, necrosis, and high cellularity tissues. With tumor margin tissues, high levels of NAA, a known marker of normal neuronal function, were observed. On the other hand, necrotic tissue presented high levels of lipids, where NAA concentrations were almost negligible and tCho was very low. High cellularity tissue expressed high levels of tCho, with corresponding low levels of NAA and lipids. Applying these models to grade III cases, two out of seven cases correlated with the tumor margin tissue model and five correlated with the high cellularity model, and all were confirmed by histopathology. For grade II tumors, 11 cases highly correlated with the high cellularity model, five cases with the tumor margin tissue model, and two cases with the necrotic tissue model, where with the nine cases validated with histopathology, agreements were seen with seven of them [23]. These results reflect the interconnectedness of metabolite behavior, emphasizing the importance of evaluating the complete metabolomic profile instead of single metabolites, and they also demonstrate the ability of HRMAS to provide greater understanding of histopathology and cell behavior.

The utility of HRMAS for the assessment of therapy responses was studied by identifying and measuring tumor biomarkers following treatment in an effort to address the limitation of MRI in differentiating tumor progressions from treatment effects in patients with GBM. Elevations in Cr, *m*-Ino, and NAA were seen in tissue surgical samples from epilepsy patients of significant astrocytosis without tumor, suggesting these metabolites as potential metabolic markers for differentiating tumor from reactive astrocytosis. Based on this observation, image-guided biopsy cores were acquired to associate *ex vivo* biochemical and pathological properties with *in vivo* MR anatomical images. After analysis of metabolomic profiles, the ratio of metabolic concentrations of *m-*Ino to tChol were found to differentiate with statistical significance between tumor, non-tumor, and treatment-induced reactive astrocytosis in both newly diagnosed and recurrent GBM [24]. The effects of 5-aminolevulinic acid (5-ALA) on GBM, in particular on the tumor-initiating cells (TICs) in the tumor margin, have also been evaluated with HRMAS MRS. GBM, as the most aggressive adult tumor, typically recurs within 2 cm of the resected tumor sites, in spite of chemotherapy and radiotherapy following surgery. The peri-operative fluorescence agent 5-ALA, capable of fluorescing tissues according to their pathological status (necrotic material and tumor margin are non-fluorescent while tumor is fluorescent), is currently used to assist the definition of tumor margins during surgery to improve surgical outcomes. TICs’ metabolomic profiles obtained from PCA evaluations of HRMAS ^1^H MRS data acquired from 10 5-ALA + *vs.* 10 5-ALA − GBMs presented no significant difference in metabolite levels, thus suggesting the inertness of the agent towards the measurable GBM metabolism [25].

Therapy effectiveness in an animal model study has also applied HRMAS as an evaluation tool. The effects of treatment with two glycolipid derivatives, a glycoside and its thioglycoside analogue, in either 20 or 40 µM concentrations on the HRMAS MRS metabolomic profiles of intact glioma C6 cells and tissues from tumor-bearing nude mice implanted with C6 glioma cells were studied. HRMAS MRS of cells treated with a 20 µM concentration of thioglycoside showed only slight changes in metabolic profile from those measured from the control, untreated cells, while cells treated with a 40 µM concentration of both thioglycoside and glycoside showed a two-fold increase in tCho. Specifically, Cho and PChol increased two and three times for each derivative, respectively, and there was also a significant rise in the ratio of PChol/GPC (1.45 ± 0.61 in treated *vs.* 0.70 ± 0.33 in the control). In intact tissue experiments, only tumors treated with 40 µM of thioglycoside showed a significant reduction in size, which also showed a two-fold increase in Cho and a 1.4-times increase in PChol with a higher PChol/GPC ratio (1.69 ± 0.68 *vs.* 1.34 ± 0.5) in treated tumors compared with those of the control. Significant increases in acetate and lactate were also seen with increasing antitumoral doses. These results mirror those acquired from cells, while tumors treated with glycoside showed metabolomic profiles similar to control samples. Overall, results indicate that glycolipid derivatives alter phospholipid metabolism, resulting in cell death [26].

Metabolomic profiles acquired with HRMAS MRS have also been used to advance basic research with the purpose of understanding the biochemical pathways related to cancer and its progression. One such study used quantitative real time polymerase chain reaction (PCR) to profile the transcripts of 18 histone deacetylases (HDACs) followed by HRMAS ^1^H MRS for the evaluation of metabolomic profiles of 15 non-tumor tissue samples from epilespy patients and 50 brain tumor patients diagnosed with grade II (*n* = 9) and grade III (*n* = 22) oligodendrogliomas, and GBM (*n* = 19). HDACs help regulate gene transcription by removing acetyl groups from lysine (Lys) amino acids on histones to allow for closer DNA wrapping. Analysis of complete metabolomic profiles with PLS-DA found that as the tumor grade increased, the Gly and the Gly/*m*-Ino ratio also increased, whereas the *m*-Ino, Cr, and the GPC/PChol ratio decreased. Patients of oligodendrogliomas with high HDAC1 expression formed a metabolically distinct group, with significantly reduced GPC/PChol ratios, from patients with unchanged HDAC1 expression. Interestingly, the GPC/PChol ratio was also significantly correlated with survival time among glioma patients, and all patients with GBM showed high HDAC expression [27]. These results suggest that the GPC/PChol ratio is associated with high-grade tumors and that the expression of HDAC1 might influence tumor aggressiveness, as reflected in the metabolomic profiles.

Brain tumor tissue HRMAS MRS studies have also contributed to the development and improvement of the methodology. One early study evaluated the effect of temperature on profiles of 43 brain tumor biopsies from patients with both meningioma (*n* = 20) and GBM (*n* = 23). Samples were frozen after collection, measured with HRMAS at both 0 °C and 37 °C, and profiles were analyzed with both PCA and linear discriminant analysis (LDA). Temperature-dependant spectral changes were observed from mobile lipids and choline-containing compounds and were mostly reversible, with the maximum non-reversible increase of 12.6% ± 7.4% for meningioma (3.22 ppm resonance) and 10.6% ± 5.7% for GBM (3.21 ppm resonance). Changes were also calculated for the 1.29/3.03 ppm ratio from single-pulse HRMAS spectra at different temperatures between 0 and 43 °C. No changes were observed between 0 and 7 °C, while above 7 °C, the ratio steadily increased with temperature. After the temperature experiment, the original spectrum at 0 °C was recovered, confirming the reversible nature of these temperature-dependent changes [28]. Another study used ^1^H and ^31^P HRMAS MRS with the application of the electronic reference to *in vivo* concentrations (ERETIC) synthetic signal to determine pH and metabolite concentrations of 33 brain tumor biopsy samples. By correlating the ^1^H-^1^H homonuclear and the ^1^H-^31^P heteronuclear experiments, direct measurement of the ^1^H-^31^P spin systems for signals that are clouded from overlapping in the ^1^H spectra was achieved. The metabolomic profiles acquired showed characteristic low levels of energetic molecules and an increased concentration of protective metabolites; however, these characteristics were strongly correlated with the amount of living tissue rather than the percent of tumor, as determined by changes in metabolites levels over sampling times of multiple hours, which could indicate the important contribution of sampling conditions on measured profiles [29]. An additional study explored the use of multivariate pattern recognition methods to generate statistical models from metabolomic profiles of 53 low-grade glioma tissue samples for prediction of aggressive tumor biology and poor clinical outcome. Cho, 2HG, *m*-Ino, and hypo-Tau were identified among the 40 highest-ranking features by the three association techniques employed: chi-squared statistics, information gain ratio, and conditional probability-based association. Recurrent low-grade gliomas that transformed into a higher grade could be distinguished from those that remained at grade II with the following metabolites: *m*-Ino, 2HG, hypo-Tau, Cho, GPC, PChol, glutathione, and lipids. On average, it was reported that gliomas that transformed into a higher grade had 56% lower levels of *m*-Ino. Hypo-Tau, glutathione, Ala, Cho, as well as an increase in activity in the hard-to-quantify range of 3.79 ppm, could differentiate between recurrent low-grade gliomas that transformed to GBM from those that transformed to grade III [30]. These studies suggest possible improvements to collection, experimental methods, and statistical methods that could advance the understanding of metabolomic profiles and their relation to cancer biology.

## 3. Breast Cancer

Breast cancer is the most diagnosed cancer in women and the second most common cancer, with an estimated 1.67 million new cases, or 25% of all cancer diagnoses, in 2012. It is also responsible for a fifth of all cancer deaths with an estimated 522,000 deaths in 2012 [1].

HRMAS MRS has been used to measure metabolomic profiles of intact breast tissue from biopsies and surgical samples. Analyzing HRMAS MRS data with the orthogonal projections to latent structure-discriminant analysis (OPLS-DA) multivariate model on 31 breast tissue biopsy cores (13 cancer, 18 non-cancer) reported 69% sensitivity, 94% specificity, and 84% accuracy in distinguishing cancer from non-cancer samples [31]. Metabolomic data of cancerous tissue showed increased levels of taurine- and choline-containing compounds when compared with benign tissue. The result of tissue metabolomic profiles obtained with the OPLS-DA model also seemed to predict the progesterone receptor (PR) status based on a training- and testing-cohort design. Out of 13 samples in the testing cohort, 10 were predicted correctly. Predicting breast cancer genotype status, such as PR, is known to have significant clinical implications. The most significant genotype statuses for human breast cancer are those of triple (estrogen receptor, ER, PR, and human epidermal growth factor receptor-2/neu, HER-2/neu) negative breast cancer (TNBC) *vs.* triple positive breast cancer (TPBC). TNBC, which lacks the expressions of ER, PR and HER-2/neu and accounts for 15%–20% of breast cancer cases, is considered the most severe and difficult-to-treat disease as it is unresponsive to many hormone-based therapies because of their lack of receptors. Metabolomic profiles obtained from PLS-DA of HRMAS MRS data acquired from 106 biopsies of 73 patients were able to differentiate between TNBC and TPBC with 77.7% accuracy (*p* = 0.001). TNBC presented increased levels of Cho and GPC and decreased levels of Cr compared with TPBC. PLS-DA was also able to distinguish hormone receptor status in tissue with similar accuracy as reported by previously discussed study [31]*.* The models showed clear separation between ER^neg^ and ER^pos^ with 72.2% accuracy (*p* < 0.001) and PR^neg^ and PR^pos^ with 67.8% accuracy (*p* < 0.001). ER^neg^ and PR^neg^ tumors showed higher levels of Gly, Cho, and Lac when compared with hormone receptor positive tumors [32].

**Figure 2:**
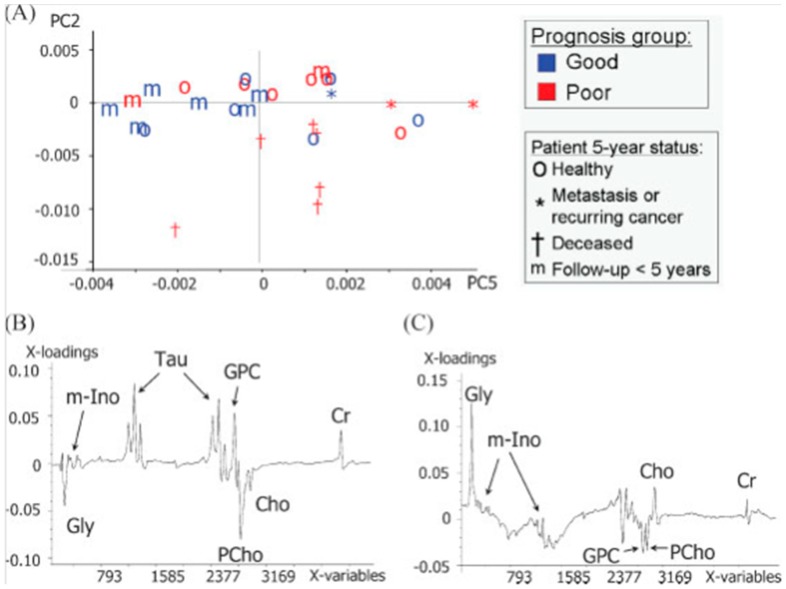
Metabolomic profiles of 29 surgical samples of palpable breast cancer correlated to patients’ prognosis and health status after five years post-surgery leading to: (**A**) Score plot for PC2 and PC5; (**B**) loading plot of PC2; and (**C**) loading plot of PC5 resulting from a PCA of region 3.6–2.9 ppm of the area normalized MR spectra (*n* = 29). In the score plot, samples from patients with different prognosis are identified by different colors (red and blue), where patient status five years after surgery is identified by different signs. PC2 accounts for 18% of spectral variation, whereas PC5 accounts for 6% [33].

HRMAS MRS–derived tissue metabolomic profiles have also been evaluated for their predicting ability of long-term disease course and prognosis for patients. A study of PCA-derived metabolomic profiles of 29 surgical samples of palpable breast lesions has identified metabolites correlated to patients’ prognosis and health status five years post-surgery [33]. Increased levels of Tau, GPC, and Cr with decreased levels of Gly and PChol have been seen with patients reported to be healthy five years post-surgery (Figure 2). The importance of evaluating a complete metabolomic profile instead of correlating only individual metabolites is again illustrated with its significantly better ability to predict prognosis than individual metabolites.

In addition to its potential to function as a diagnostic tool, HRMAS has been increasingly used to measure and assess the therapy effects of anticancer drugs. One drug target of interest has been the phosphatidylinositol 3-kinase (PI3K) pathway, since it is frequently activated in cancer cells through mutations. Inhibitors that target parts of the PI3K pathway have the potential to produce promising results for patients with activated PI3K pathways. Experimental tissues from xenograft models representing both basal-like and luminal-like breast cancer have been used to quantify the PI3K pathway associated metabolite changes before and after treatment with PI3K inhibitors (MK-2206 and BEZ235), and to determine metabolic biomarkers that reflect treatment response. Both inhibitors reduced Lac and increased PChol levels in basal-like tumors. BEZ235 increased glucose and GPC in basal-like tumors as well. The inhibitors produced no measurable changes to the metabolomic profiles of the luminal-like cancers [34]. A HRMAS ^31^P MRS study on the effect of the PI3K inhibitor BEZ235 with xenografts of both basal-like and luminal-like breast cancer was carried out to objectively quantify phosphorylated metabolites in intact cancer tissue. The ^31^P MRS spectra showed clear differences between the basal-like and luminal-like breast cancer tissue, with a distinct inverse relationship between PChol and GPC levels showing PChol elevated in luminal-like samples and GPC elevated in basal-like samples. BEZ235 treatment in basal-like xenografts led to a significant decrease in PE of −25.6% (*p* = 0.01) as well as a 16.5% increase in PChol (*p* = 0.02) and a 37.3% increase in GPC (*p* < 0.001) [35]. Since basal-like breast cancer is typically characterized by a lack of hormone receptors and poor prognosis, measurements of HRMAS results on drug effects could assist the selection of appropriate targeted therapies and facilitate their translations into clinic.

Genetic models that are either sensitive or resistant to drug treatments have been studied for their metabolomic responses resulting from resistance and incomplete treatment with docetaxel, a common chemotherapy drug for breast cancer. During the study, HRMAS ^1^H MRS was performed on biopsy cores from BRCA 1–mutated mammary tumors in mice that were either docetaxel-sensitive or -resistant. PCA distinguished between the resistant and sensitive tumors before treatment. The resistant tumors showed higher levels of all choline groups whereas the sensitive tumors had higher levels of Gly, Tau, and Cr. With docetaxel treatment, the choline compounds increased in sensitive tumors, but remained stable in resistant tumors, identifying Cho as an earlier biomarker for docetaxel response [36].

**Figure 3:**
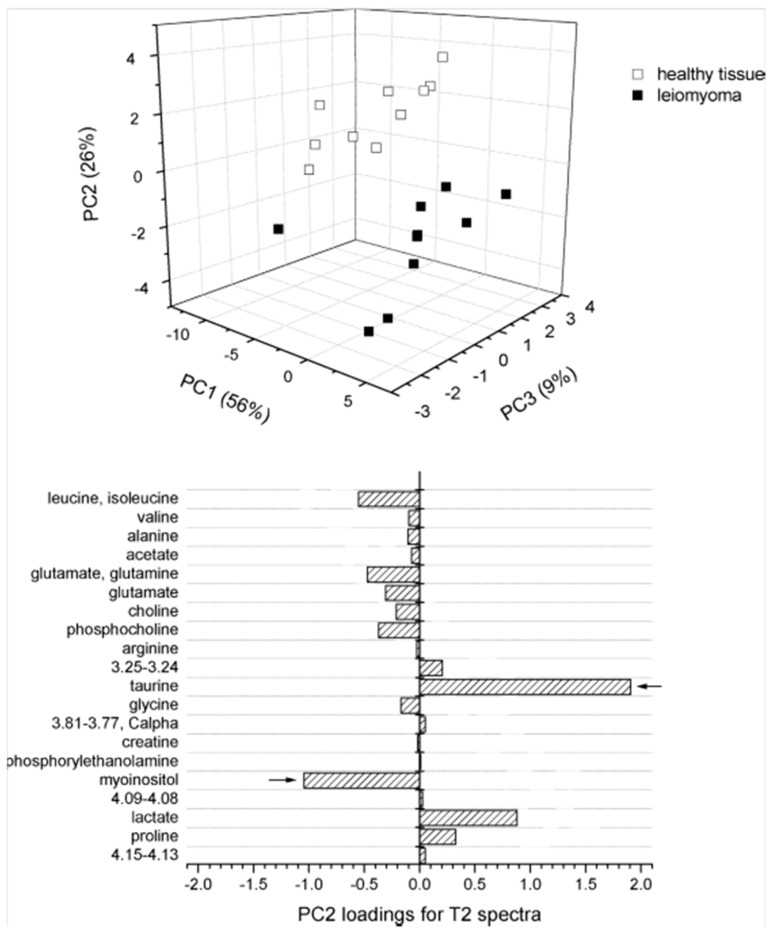
PCA score plot (top) and loading plot for PC2 (bottom) from ^1^H HRMAS NMR t_2_-edited spectra for comparison between healthy myometrium and leiomyoma uterine tissues [37].

Metabolomic profiles acquired with HRMAS MRS have also been used to characterize other cancers of the female reproductive system, including uterine and ovarian cancers. Using both 1D and 2D HRMAS MRS, the metabolomic profiles of healthy myometrium and benign uterine tumors, leiomyoma were compared. When spectra were acquired using a Carr-Purcell-Meiboom-Gill (CPMG) to enhance spectral resolution, PCA was clearly able to differentiate between myometrium and leiomyoma (Figure 3) [37]. Using both *in vivo* MRI/MRS and *ex vivo* HRMAS MRS, characterization of epithelial ovarian carcinoma (EOC) was attempted with tissue obtained by implanting SKOV3.ip cells into severe combined immunodeficiency mice [38]. When compared to *in vivo* MRS, profiles acquired *ex vivo* with HRMAS showed higher variability, likely due to tissue heterogeneity existing in the large tumor masses. While the low resolution *in vivo* MRS spectra could not quantify Tau, *s*-Ino, or Ala levels, the observations of these metabolites with HRMAS spectra did not show significant variations. HRMAS profiles confirmed that PChol was the major contributor of the tCho signal. EOC has the highest death rate among all gynecological malignancies, for typically it presents no symptoms until its spread to the abdominal cavity, and patients frequently relapse and experience drug resistance. Utilizing both HRMAS MRS and the applicability of xenograft tissue could provide insight into EOC disease progression, and serve as a preclinical tool for understanding biochemical changes in response to drug therapy.

## 4. Prostate Cancer

Prostate cancer is the second most frequently diagnosed cancer in men, with an estimated 1.1 million new cases worldwide, representing 15% of new cancer cases in men, in 2012. It is also the fifth-highest cause of cancer-related death in men, resulting in 307,000 estimated cancer deaths in 2012 [1]. An elevated prostate-specific antigen (PSA) level detected during an annual serum screening test typically indicates the need for a prostate biopsy. Since the advent of PSA testing, prostate cancer has been increasingly diagnosed at early stages, when lesions are small and distributed randomly throughout the prostate. The small size and dispersal of lesions has led to both false-negative biopsies, and overtreatment for the current histopathology of biopsy cores, relying mostly on the Gleason score system, is insensitive in differentiating aggressive from indolent disease for the majority of cases detected through PSA testing. To overcome those clinical challenges, research using HRMAS MRS has focused its efforts not only on characterizing benign *vs.* malignant prostate tissue, but also, more importantly, on identifying aggressive *vs.* indolent disease.

HRMAS ^1^H MRS has been applied to evaluate the occurrence, amount, and aggressiveness of cancer with 149 human prostate tissue samples from 40 patients. While analysis of malignant tissues did not find any specific ratio between metabolites that could correlate to the Gleason score, evaluations for non-malignant tissue taken at various distances from the tumor loci revealed that the ratios of *m*-Ino/*s*-Ino and Chol/Cr could be significantly related to the Gleason score of the tumor and could differentiate between tumors with Gleason scores of 6 and 7. Acknowledging the commonly observed fact that a prostate biopsy core usually contains a mixture of malignant and non-malignant tissue, the study further used multivariate linear regression to determine the fraction of cancerous tissue in a sample. The reported results show that the mean ratios of GPC and PChol/Cr, both *m*-Ino/*s*-Ino, Cho/Cr, and *s*-Ino/Cr correlated significantly (*p* < 0.05) with the tumor fraction of the sample; and the GPC and PChol/Cr also correlated significantly (*p* < 0.001) with the expression levels of the cell proliferation marker Ki67 and differentiated between malignant and non-malignant tissue [39].

Realizing the clinical challenge in early identification of prostate cancer cases that possess the potential to recur, retrospective HRMAS MRS evaluations of prostate cancer metabolomic profiles have been reported. Metabolomic analysis of 16 cases of recurrent prostate cancer, defined as increases in serum PSA levels after prostatectomy, was carried out with two groups of 16 paired and randomly selected cases without recurrence [40]. PCA analysis was conducted on the 27 most intense spectral regions selected based on the threshold above the median of all peaks. Four principle components (PCs) that correlated with tissue pathological features were identified for further canonical analysis between the recurrent cases and their first group of 16 matched non-recurrent cases. Applications of results thus obtained from the second group of the other 16 matched non-recurrent cases revealed the metabolomic predictive power of prostate cancer recurrence to be at the level of 78% accuracy, significantly improved from the current ability of the clinical paradigm at the level of 50% when evaluating prostate cancer recurrence potential for matched cases.

In addition to intact tissue studies, HRMAS MRS has also been used to acquire metabolomic profiles of cancer cell lines , including PC3, for their response to chemotherapy [41]. For instance, the metabolomic profile of PC3 cancer cells showed increased PChol, Lac and fatty acids, and decreased acetate and cellular amino acids (Gln, proline (Pro), Asn, Arg, Gly, Ala, Lys, phenylalanine (Phe), and leucine (Leu)). These results suggest that HRMAS could be used to characterize cellular metabolic pathways, as well as provide insight into tumor cell phenotypes.

## 5. Lung Cancer

Lung cancer is the leading cause of cancer death, representing 19.4% of all cancer-related deaths with 1.6 million deaths, and has the highest incidence rate, representing 12.9% of all new cancer cases in 2012 with 1.8 million people diagnosed worldwide [1]. In about 80% of lung cancer patients, symptoms at the early stage of the disease are not diagnosed until the late stage due to a lack of effective screening tools. Metabolomic analyses for lung cancer with HRMAS MRS have been evaluated as a potential diagnostic and characterization tool for future clinic use.

Several studies have attempted to use HRMAS MRS to determine metabolomic profiles of lung cancer [42]. A 2010 study assessed the ability of HRMAS ^1^H MRS to differentiate between 24 paired tumor and adjacent benign samples, as well as between profiles from different tumor types. When PCA was applied to the whole spectra (from 0.5 to 8.75 ppm), most tumor tissues were located in negative PC1 and all adjacent tissues were located in positive PC1, showing a clear difference in the profile between cancer and benign tissue, with Lac, GPC, PChol, Tau, Cr, and lipids appearing elevated in tumors, while glucose, acetate, and methionine (Met) appeared elevated in benign tissue. The score plots of PLS-DA results show strong separation between adenocarcinomas (*n* = 12), carcinoid tumors (*n* = 4), and epidermoid carcinomas (*n* = 4), with a strong negative correlation for GPC, Tau, ascorbate, and some broad signals most likely arising from oligopeptides [43]. A similar study confirmed the ability of HRMAS ^1^H MRS to differentiate between benign and malignant lung tissue, and agreed, through PCA analysis, that Lac, PChol, and GPC levels are elevated in tumors, while acetate, *m*-Ino, inosine/adenosine, and glucose are reduced [44].

**Figure 4:**
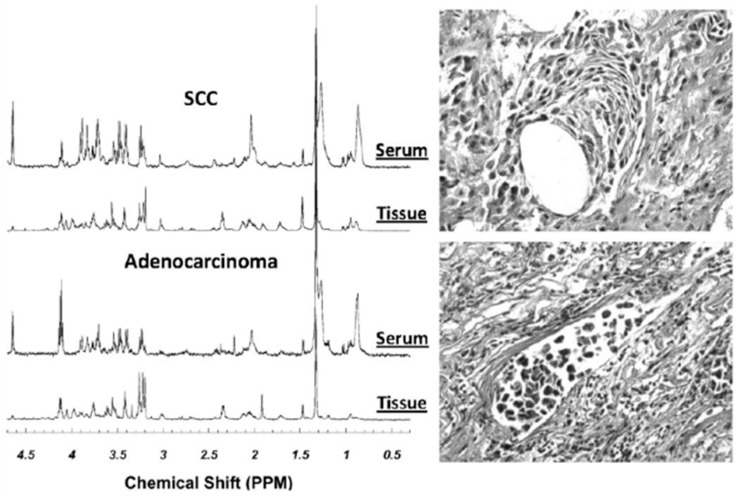
Examples of HRMAS MR spectra of intact tissue and paired sera from the same patients with either SCC or AC of the lung. The tissue histopathology images obtained from the tissue samples after spectroscopic measurements are also presented. From these images, tissue pathologies were quantified [45].

Other studies have attempted to pair metabolomic profiles of lung cancer tissues with serum samples in the hopes of developing a much-needed diagnostic blood screening tool for large populations. These attempts were stimulated by the fact that while CT can detect small lung lesions of early stage cancer, its high cost coupled with the potentially hazardous dose of radiation for general populations precluded its use as a diagnostic tool of yearly screening tests for lung cancer. Thus, establishing serum metabolomic profiles for lung cancer potentially offers a triage process to screen suspicious patients with CT for cancer detection at early, asymptomatic stages. Paired tissue and serum samples from 14 patients with squamous cell carcinoma (*n* = 5) and adenocarcinoma (*n* = 9), as well as serum from seven healthy control subjects, were analyzed using HRMAS ^1^H MRS followed by tissue quantitative histopathology [45]. PCA was performed on both tissue and serum for 21 spectral regions identified from spectra of tissue. As in the previously reported studies, tissue metabolomic profiles were able to differentiate between squamous cell carcinoma and adenocarcinoma (Figure 4). When these profiles were applied to serum spectra, serum profiles of cancer and healthy controls could be differentiated with statistical significance through nominal logistic analysis (*p* < 0.0001). Despite the small sample size, this observation strongly supports the further investigations and future utility of serum metabolomic profiles as a screening tool for lung cancer, with the potential to increase early diagnosis and treatment of patients.

## 6. Cancers of the Gastrointestinal Tract

Colorectal cancer (CRC) is the third most common cancer in men, resulting in 746,000 new cases or 10%, and the second most common cancer in women, with 614,000 new cases or 9.2%, worldwide in 2012 [1]. It is also responsible for 8.5% of all cancer deaths, with 694,000 deaths in both sexes worldwide in 2012. Currently, tests for early diagnosis, which include fecal occult blood testing (FOBT) and colonoscopy in addition to biopsy with subsequent histopathology, cannot provide any biochemical insight into tumor prognosis.

Metabolomic profiles from intact tumor samples and adjacent tissue have shown abilities in distinguishing malignant from adjacent non-cancer tissue, and differentiating between tumors of different T- and N-stages based on TNM classification, where T describes the size of the original tumor, N describes nearby involved lymph nodes, and M describes distant metastasis. Metabolomic profiles obtained from OPLS-DA of HRMAS MRS results acquired from 83 intact tumor samples and 87 samples of adjacent mucosa from 26 patients undergoing resection for CRC showed abilities of differentiating between malignant and adjacent samples. In tumor samples, contributions from Tau, isoglutamine, Cho, Lac, Phe, and tyrosine (Tyr) were increased, and those from lipids and triglycerides were decreased, as seen in the profiles. Metabolomic profiles from tumor-adjacent mucosa located 10 cm away from the tumor margin were able to predict five-year survival with higher accuracy than those measurable from tumor tissue. Lower concentrations of iso-butyrate, acetate, and Cho were observed in the adjacent tissue of patients who were healthy five years post-surgery compared to patients who did not survive [46]. Similar findings were reported by another study of analyzing the metabolomic profiles of 171 surgical tissue samples from 44 patients (center of tumor, *n* = 88; 5 cm from tumor margin, *n* = 83). The study also reported higher levels of acetate (*p* < 0.005) and Arg (*p* < 0.005) and lower levels of Lac (*p* < 0.005) in colon cancer samples (*n* = 49) when compared with rectal cancer samples (*n* = 39) [47].

HRMAS has also been employed to characterize human esophageal cancer. Esophageal cancer is typically diagnosed through endoscopy, yielding approximately 20 biopsy cores for histological evaluation. While biopsy is considered the current diagnostic standard, such random biopsies always carry the possibility of missing the cancer lesions and resulting in false negatives. To improve diagnostic probability, metabolomic profiles have been investigated for their ability to differentiate histologically normal mucosa from esophageal cancers and non-diseased controls. HRMAS MRS was used to acquire metabolomic profiles from 35 patients with esophageal adenocarcinoma and 52 age-matched healthy controls. Analysis with PCA in the region from 0.75 to 5.6 ppm show that 26% and 12% of the metabolomic profile was found to be unique to the tumor or control tissue, respectively. PChol, Glu, *m*-Ino, adenosine-containing compounds, and inosine exhibited significant differences that distinguish the two groups. Of note, the PChol/Glu ratio in histologically normal tissue signified the presence of esophageal cancer (*n* = 123; *p* < 0.001), suggesting that this ratio could serve as a diagnostic biomarker [48]. HRMAS MRS–derived metabolomic profiles of esophageal tumors were also used to assist in characterization of tumor differentiations. Increased levels of Glu and Ala and decreased levels of Cr and Tau were observed in moderately differentiated tumors compared with well-differentiated tumors [49].

A nude mouse pancreatic cancer xenograft model created by subcutaneous injection of human pancreatic cancer cell SW1990 was investigated by HRMAS MRS, which generated metabolomic profiles for the treatment effect of radiotherapy. Profiles generated from PCA of HRMAS MRS data showed increased expression levels for Cho, Tau, Ala, isoleucine (Ile), Leu, valine (Val), Lac, and Glu and decreased levels of PChol, GPC, and betaine for pancreatic cancer tissues compared with the normal nude mouse pancreas. While there was no visible change in the metabolomic profile after treatment with three different radiation doses of 10, 20, and 30 Gy, PCA revealed a decrease in Cho and betaine and an increase of acetic acid with increased radiation dose [50]. Tissue HRMAS MRS has also been used to measure the effect of brivanib, a dual Tyr kinase inhibitor that targets angiogenesis receptors, on hepatocellular carcinoma xenografts. Results show a significant difference in metabolomic profiles between tissue from the tumor-suppressed treated group and the control, represented especially by a decrease in choline groups in the treated group, suggesting the functions of brivanib in decreasing cell proliferation and increasing apoptosis [51].

## 7. Cancers Relating to the Neuroendocrine Systems

**Figure 5:**
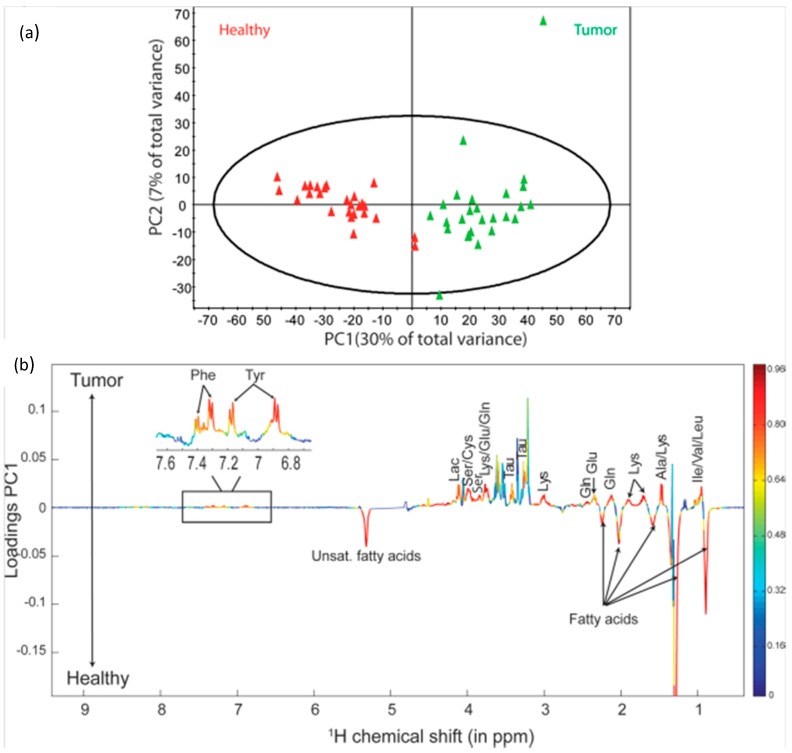
(**a**) PCA score plot showing the discrimination between thyroid lesions and their healthy counterpart tissues; (**b**) PC1 loadings plot showing the model coefficients for each NMR variable. Horizontal axis corresponds to the NMR chemical shift scale; vertical axis corresponds to the variable weights on PC1. The line variation corresponds to model covariance derived from the mean centered model, whereas the color map corresponds to the correlation coefficient derived from the unit-variance model. Significantly discriminant metabolites were annotated on the model coefficient plot [52].

A substantial amount of metabolomic research has been focused on various tumors originating in the neuroendocrine systems. One such area of interest is the thyroid. Thyroid nodules are common problems in the clinic, where one of the main concerns is to rule out thyroid cancer. Metabolomic profiles acquired with HRMAS MRS were able to distinguish between healthy tissue and thyroid lesions, and differentiate tumors based on malignancy grade. Spectral profiles from healthy thyroid tissue and thyroid lesions were clearly distinguishable by visual comparison, as well as the results of PCA, with the loading factor for PC1 indicating that the differentiation could be attributed to an increase in Lac and other amino acids (Phe, Tyr, serine (Ser), Lys, Tau, Gln, Glu, Ala, Ile, Leu, and Val) as well as a decrease in lipids in tumor samples (Figure 5). PCA results were unable to discriminate between malignant and benign tumor samples; however, analysis with OPLS-DA indicated the possibility for differentiating between the two groups (*p* = 4.10^−4^) with an increase in Lac and Tau and a decrease of Cho, PChol, *m*-Ino, and *s*-Ino in malignant samples, as shown by the loading factors [52,53].

Studies have also explored the possibility of utilizing metabolomic profiles to differentiate between healthy and malignant glandular tissue. Metabolomic profiles for the normal adrenal cortex and medulla compared with those of adenoma, adrenal cortical carcinoma, and pheochromocytoma have been assessed recently. PLS-DA results showed a separation between the normal adrenal cortex and normal medulla, with the normal medulla characterized by significantly higher levels of adrenaline, noradrenaline, *m*-Ino, Gln, glutathione, Lac, Cr, *s*-Ino, Gln, Tau, Gly, Cho-containing metabolites, and succinic and ascorbic acids. Separations were also seen between metabolomic profiles of the normal adrenal cortex and adrenal cortical carcinoma, normal adrenal medulla and pheochromocytoma, and adrenal cortical carcinoma and adenoma according to the results of PLS-DA. Profiles of adrenal cortical carcinoma were reflective of malignant tissue, with increased choline compounds suggesting higher phospholipid turnover, various amino acid resonances suggesting an increase in production, and metabolites indicative of anaerobic processes and increased glycolysis such as Lac. Despite these clear results, adrenal cortical carcinoma tissue samples from patients with aggressive disease (metastatic spread at diagnosis or within one year; *n* = 7) could not be separated from those without metastasis (*n* = 5) even with PLS-DA analysis, due likely to the small sample size of the study [54].

In addition to differentiating between healthy and malignant tissue, metabolomic profiles have been used to differentiate between various mutations of cancer, aimed at guiding treatment and determining prognosis for patients. Results from a study were able to differentiate between sporadic (*n* = 48) and succinate dehydrogenase gene (SDHx) mutation-related (*n* = 23) pheochromocytomas/ paragangliomas by analyzing the 24 identified metabolites present in all 71 spectra with PCA and OPLS-DA. Through these analyses, 12 metabolites were identified, showing SDHx-related pheochromocytomas/paragangliomas could be characterized by higher levels of succinate, *m*-Ino, Met, Gln, Tau, and ATP while sporadic tumors were characterized by higher levels of adrenaline, noradrenaline, Glu, glutathion, ascorbate, and Asp [55].

PCA and OPLS-DA have also been applied to differentiate HRMAS MRS metabolomic profiles of cells with six pathogenic missense Menin variants from those with functional and over-expression of WT Menin. Mutations to the *MEN1* gene, which encodes the Menin protein, are responsible for multiple endocrine neoplasia syndrome, type 1 (MEN1), an example of a hereditary cancer. The results of the study identified decreases in eight major contributing metabolites responsible for the differentiations: PChol, Cho, Tau, Cr, aspartate, glutathione, ƴ-amino-*N*-butyrate, and inosine [56]. Development of diagnostic tools to identify mutations is critical for accurate diagnosis, treatment, and evaluation of familial risks. These studies present the possibility of utilizing HRMAS to classify tumors by their genetic background, thereby improving the ability to accurately diagnosis and target treatment.

## 8. Emerging Research Directions

The major benefit of HRMAS MRS as a method for characterization of tissue is its preservation of tissue structures for subsequent histological and molecular analyses. Several studies have investigated the integrity of the mRNA of intact tissues after their HRMAS MRS studies for gene expression evaluations in the attempt to link metabolomics with transcriptomics. Results from a comparative study between 21 prostate tissue samples (*n* = 11 biopsy, *n* = 10 surgical) that experienced HRMAS MRS measurements and a control group of 19 tissue samples (*n* = 9 biopsy, *n* = 10 surgical) that bypassed MRS measurements showed no significant difference in terms of histological and mRNA integrities [57,58]. Following conversion to cDNA and subsequent analysis of microarrays, there was no significant difference in over- and under-expressed genes between HRMAS and the control samples, indicating that HRMAS MRS does not prevent subsequent genetic microarray analysis, and the RNA was still intact after HRMAS MRS to provide correlations between transcriptomic and metabolomic profiles of the same surgical samples. In 2012, this combination concept was applied to create a machine learning–based data fusion framework that integrated heterogeneous data sources of metabolic and molecular datasets, acquired through techniques such as HRMAS MRS and gene transcriptome profiling, to classify different brain tumors [59]. The framework works by identifying certain biomarkers from each data set to use as classifiers for five different categories of brain tumor biopsies: 11 GBM, eight anaplastic astrocytoma, seven meningioma, seven schwanoma, and five adenocarcinoma. The results outperformed any individual dataset in classifying tumors.

Since metabolomics, which is similar to genomics and proteomics, involves evaluations of large ensembles of data sets, the successes in the establishment of cancer metabolomics rely significantly on the execution of correct data analytical protocols from various statistical methods. From the metabolomic results reviewed above, the long list of statistical protocols can be appreciated, including *t*-test, ANOVA, multivariate linear regression, PCA, LDA, PLS-DA, OPLS-DA, *etc*. Considering the structure of metabolomic data sets, which are often presented as having larger numbers of metabolic variables than the number of cases, and are always affected by known or unknown biological variables, special cautions need to be exercised when analyzing these data sets. For instance, PCA, an unsupervised statistical method, can function to effectively reduce data dimensions and is a valuable tool to resolve the issue of variable numbers greater than case numbers often seen with metabolomic data sets. Nevertheless, evaluations of principal components thus obtained, similar to the analyses of individual variables, with *t*-test, ANOVA, regressions, *etc.* should also avoid the possibility of false-positives due to type I errors of multiple comparisons. On the other hand, when supervised statistical methods, such as various discriminant models, are employed in the data analyses, the observed group separations directly resulting from discriminant analysis should not stir too much excitation and rushing to the declaration of success should be avoided. Without testing the results generated from discriminant models with another independent group (a testing cohort), the observed group separations seen (in a training cohort) directly from a discriminant analysis can only at best be considered as a proposal of hypotheses, since a discriminant analysis model is designated to produce such a separation through data manipulations even if the real separation is not in existence. Incorrect applications of any statistical method can produce false-positive or false-negative results that not only can mislead the clinic, but can also derail the scientific development and public acceptance of cancer metabolomics.

## 9. Conclusions

Analysis of metabolomic profiles acquired with *ex vivo* HRMAS MRS can significantly contribute to understanding and characterizing cancer disease and progression (see Table 1 for summary of metabolite changes related to different cancer types), as well as informing treatment response. With continually improving methodology, HRMAS MRS presents strong potential to enhance diagnostic measures in the clinic, providing additional information about individual cancer biology and prognosis to help inform care for both clinicians and patients.

## Figures and Tables

**Table 1:** Summary of observed metabolite changes after high resolution magic angle spinning (HRMAS) magnetic resonance spectroscopy (MRS) in response to cancer type.

Cancer	Reference	Observed Metabolic Changes
**Brain**	[14]	*Astrocytoma, grade II:* glyerophosphocholine (GPC)↑, myo-Inositol (*m*-Ino)↑ *Glioblastoma Multiforme (GBM):* phosphocholine (PChol)↑, glycine↑, lipids↑
	[16]	*Glioma, grades II–IV:* progressive reduction in *m*-Ino to total choline (tCho) index
	[17]	*Hemangiopericytoma (HPC) compared with meningioma: m*-Ino↑, glucose↑, gluthatione↑, creatine (Cr)↓, glutamine↓, alanine (Ala)↓, glycine (Gly)↓, choline (Cho)↓, PChol↓, GPC↓
	[18]	*Meningioma, grades I–III:* progressive Ala↓and Cr↓
	[19]	*Biomarkers of GBM vs. metastasis:* Cr, Gly, glutamine (Gln), hypotaurine (hypo-Tau)
	[20]	*Astrocytoma, grade I–II vs. grade III:* N-acetyl-aspartate *(*NAA)↑, Cr↑, GPC↑, *m*-Ino↑, lactate (Lac)↓, PChol↓ *Astrocytoma, grade I–II vs. grade IV:* Lac↑, Cr↑, Cho↑, GPC↑, Gly↓, PChol↓ *Astrocytoma, grade III vs. grade IV:* Lac↑, Cr↑, Cho↑, GPC↑, *m*-Ino↓, PChol↓
	[21]	*Ependymoma: m*-Ino↑ *Medulloblastoma:* taurine (Tau)↑, GPC↑, PChol↑, Cho↑ *Pilocytic astrocytoma:* fatty acids↑
**Lung**	[44]	Lac↑, PChol↑, GPC↑, acetate↓, *m*-Ino↓, inosine/adenosine↓, glucose↓
**Thyroid**	[52,53]	Lac↑, phenylalanine (Phe)↑, tyrosine (Tyr)↑, serine (Ser)↑, lysine (Lys)↑, Tau↑, Gln↑, glutamate (Glu)↑, Ala↑, isoleucine (Ile)↑, leucine (Leu)↑, valine (Val)↑, lipids↓, Cho↓, PCho↓, *m*-Ino↓, *s*-Ino↓
**Adrenal**	[54]	*Adrenal cortical carcinoma:* tCho↑, Lac↑, glutathione↑, *m*-Ino↑, glycine↑, Cr↑, Glu↑, Gln↑, *scyllo*-Inositol (*s*-Ino)↑, NAA↓, Ile↓ *Adenoma:* succinic acid↑, Val↓, Ala↓, Asp↓, gamma-aminobutyric acid (GABA)↓, Ile↓, acetate↓, Lys↓ *Pheochromocytoma:* Tau↑, Ala↑, aspartate↑, GABA↑, glutathione↑, noradrenaline↑, ascorbic acid↑, tCho↑
	[55]	*Succinate dehydrogenase gene (SDHx) Pheochromocytoma/ paraganglioma:* succinate↑, *m*-Ino↑, Met↑, glutamine↑, Tau↑, adenosine triphosphate (ATP)↑
	[56]	*Multiple endocrine neoplasia syndrome, type 1 (MEN1):* PChol↓, Cho↓, Tau↓, Cr↓, aspartate↓, glutathione↓, ƴ-amino-*N*-butyrate↓, inosine↓
**Colorectal**	[46,47]	Tau↑, isoglutamine↑, Cho↑, Lac↑, Phe↑, Tyr↑, lipids↓, triglycerides↓
**Esophageal**	[48]	PChol, glutamate, *m*-Ino, adenosine-containing compounds, inosine
**Prostate**	[39]	*Ratios correlating with tumor fraction:* GPC+PChol/Cr, *m*-Ino/*s*-Ino, Cho/Cr, *s*-Ino/Cr *Ratio correlating with malignancy and Ki67:* GPC+PChol/Cr
**Breast**	[31]	Tau↑, tCho↑
	[32]	*Triple negative breast cancer (TNBC) as* *compared with triple positive breast cancer (TPBC):* Cho↑, GPC↑, Cr↓ *Estrogen receptor negative (ER^neg^) and progesterone receptor negative (PR^neg^):* glycine↑, Cho↑, Lac↑
	[33]	*5-year survival:* Tau↑,GPC↑, Cr↑, glycine↓, PChol↓
**Uterine**	[37]	*Leiomyoma:* glutamate↑, glutamine↑, Tau↓

## Abbreviations

The following abbreviations are used in this manuscript:

2HG	2-hydroxyglutarate
5-ALA	5-aminolevulinic acid
Ala	alanine
ANOVA	analysis of variance
Arg	arginine
Asn	asparagine
Asp	aspartate/aspartic acid
ATP	adenosine triphosphate
Cho	choline
CPMG	Carr-Purcell-Meiboom-Gill
Cr	creatine
ER	estrogen receptor
EOC	epithelial ovarian carcinoma
ERETIC	electronic reference to *in vivo* concentrations
FMW	focused microwave
FOBT	fecal occult blood testing
GABA	gamma-aminobutyric acid
GBM	glioblastoma multiforme
Gln	glutamine
Glu	glutamate/glutamic acid
Gly	glycine
Glx	glutamate and glutamine
GPC	glycerophosphocholine
HDAC	histone deacetylases
HER-2/neu	human epidermal growth factor receptor-2/neu
HPC	hemangiopericytomas
HRMAS	high resolution magic angle spinning
Ile	isoleucine
Lac	lactate
LDA	linear discriminant analysis
Leu	leucine
Lys	lysine
Man	mannitol
MCI	*m*-Ino to tCho index
MEN1	multiple endocrine neoplasia syndrome, type 1
Met	methionine
MM	macromolecule
MRI	magnetic resonance imaging
MRS	magnetic resonance spectroscopy
*m*-Ino	*myo*-Inositol
NAA	N-acetyl-aspartate
NMR	nuclear magnetic resonance
OPLS-DA	orthogonal partial least square discriminant analysis
PC	principal component
PCA	principal component analysis
PChol	phosphocholine
PCr	phosphocreatine
PCR	polymerase chain reaction
PE	phosphoethanolamine
Phe	phenylalanine
PI3K	phosphatidylinositol 3-kinase
PLS-DA	partial least squares discriminant analysis
Ppm	parts per million
PR	Progesterone receptor
PRESS	point resolved spectroscopy
Pro	proline
PSA	prostate specific antigen
RIN	RNA integrity number
*s*-Ino	*scyllo*-Inositol
SDHx	succinate dehydrogenase gene
Ser	serine
T	tesla
Tau	taurine
TIC	tumor-initiating cells
TNBC	triple negative breast cancer
TPBC	triple positive breast cancer
Tyr	tyrosine
Val	valine
WHO	World Health Organization
